# Concordance of obesity classification between body mass index and percent body fat among school children in Saudi Arabia

**DOI:** 10.1186/s12887-015-0335-6

**Published:** 2015-03-05

**Authors:** Abdulrahman Al-Mohaimeed, Saifuddin Ahmed, Khadiga Dandash, Mohammed Saleh Ismail, Nazmus Saquib

**Affiliations:** Family and Community Medicine Department, College of Medicine, Qassim University, Qassim, Saudi Arabia; Department of Population, Family and Reproductive Health and Department of Biostatistics, Bloomberg School of Public Health, Johns Hopkins University, Baltimore, MD USA; Food Science & Human Nutrition Department, College of Agriculture and Veterinary, Qassim University, Qassim, Saudi Arabia; College of Medicine, Sulaiman AlRajhi Colleges, Al Bukairyah 51941, Kingdom of Saudi Arabia, PO Box 777, Al-Qassim, Saudi Arabia

**Keywords:** Children, Anthropometry, Body mass index, BMI, Percent body fat, Saudi Arabia, Obesity, Near infrared reactance, NIR

## Abstract

**Background:**

In Saudi Arabia, where childhood obesity is a major public health issue, it is important to identify the best tool for obesity classification. Hence, we compared two field methods for their usefulness in epidemiological studies.

**Methods:**

The sample consisted of 874 primary school (grade I-IV) children, aged 6–10 years, and was obtained through a multi-stage random sampling procedure. Weight and height were measured, and BMI (kg/m^2^) was calculated. Percent body fat was determined with a Futrex analyzer that uses near infrared reactance (NIR) technology. Method specific cut-off values were used for obesity classification. Sensitivity, specificity, positive and negative predictive values were determined for BMI, and the agreement between BMI and percent body fat was calculated.

**Results:**

Compared to boys, the mean BMI was higher in girls whereas the mean percent body fat was lower (p-values <0.0001). According to BMI, the prevalence of overweight or obesity was significantly higher in girls (34.3% vs. 17.3%); as oppose to percent body fat, which was similar between the sexes (6.6% vs. 7.0%). The sensitivity of BMI to classify overweight or obesity was high (boys =93%, girls = 100%); and its false-positive detection rate was also high (boys = 63%, girls = 81%). The agreement rate was low between these two methods (boys = 0.48, girls =0.24).

**Conclusions:**

There is poor agreement in obesity classification between BMI and percent body fat, using NIR method, among Saudi school children.

## Background

The prevalence of childhood obesity in Saudi Arabia has been increasing consistently for the past 20 years. The estimate of overweight children in 1995 was around 11% [1] and recent data indicated that the prevalence was in the range of 20-25% [2]. Although the prevalence in the reported studies varied according to the age and sex distribution of their samples, a clear geographical variation in the overweight prevalence is evident [3]. The highest prevalence is reported in the central region whereas the lowest prevalence is in the southern region of the kingdom.

The prevalence of obesity is also likely to vary by its method of estimation. There are multiple methods to measure adiposity such as body mass index (BMI) [4], skinfolds [5], waist circumference (WC) [6], waist to hip ratio (WHR) [7], underwater weighing (densitometry) [8], bio-electrical impedance analysis (BIA) [9], near infrared reactance (NIR) [10], dual-energy X-ray absorptiometry (DEXA) [9], and magnetic resonance imaging (MRI) [11]. BMI is relatively easy to calculate, inexpensive, and hence is widely used in the epidemiological studies. However, it does not distinguish between fat and lean body mass. On the other hand, densitometry, DEXA, and MRI provide more accurate measures of obesity but are unsuitable for epidemiological studies since they require specialized equipment and skilled manpower; and they are expensive.

BIA and NIR are alternative field methods for measuring body fat; they both use portable machines, and their results are reproducible. Both methods have shown good correlation with DEXA, but NIR has several advantages over the BIA. NIR is not subject to variation by ethnicity, consumption of food and beverages, or exercise; and it does not require pre-test calibration.

Most epidemiological studies among children in Saudi Arabia used BMI as the method of estimation for overweight and obesity [1-3,12-14]. There is a need to compare BMI to other field methods because of its limitations; and a field method, such as NIR, may be more appropriate because it distinguishes between fat and lean body mass. There has been no study to date in this regard among children in Saudi Arabia.

Hence in a cohort of Saudi school children, we determined the overweight and obesity prevalence according to BMI and percent body fat. Further, we determined the sensitivity, specificity, positive and negative predictive value for BMI, and the agreement rate between BMI and percent body fat for overweight or obesity classification. We used established sex and age specific cut-off points for BMI [15] and percent body fat [16].

## Methods

### Overview of the study

The office of the deanship of research at the Qassim University, Saudi Arabia, funded this cross-sectional study; and the study protocol was approved by the Research Ethical Committee at Qassim University. The investigators were constrained to sample only the school children of Qassim province because of budgetary limitation. The study was conducted in 2012, and the duration of sampling and data collection was six months. The study participants were all Saudi nationals as the public schools in Saudi Arabia, from where the sample was drawn, are reserved for its citizens. Although the number of male and female students in the Saudi primary schools are comparable, the low enrollment of female participants into the study was due to insufficient female research staff.

### Sample and sampling strategy

The sample consisted of 874 primary school children (male: 618; female: 256). It was obtained through a multi-stage random sampling procedure; first, two cities (e.g. Buraydah and Unaiza) were randomly selected from a list of 10 cities in the Al-Qassim province; then a total of 34 schools (30 from Buraydah and 4 from Unaiza according to the population proportion) were randomly selected from a list of 340 schools. Finally, a class list was created for each targeted grades (from grade one to four) in the selected schools. Ten classes from each grade were randomly selected (40 classes). The average number of students per class was around 25, which meant approximately 1000 students were eligible. Being a native, having a provincial residency permit, and an age range of 6–10 years were inclusionary criteria. On the other hand, being disabled (physically or mentally), having a diagnosis of chronic disease, psychiatric illness, or immune-compromised disorder were exclusionary criteria.

All students in the selected classes were invited to participate. The students received a form to be taken to their parents at home. The form included a brief description of the study, an invitation to participate, and an informed consent document. Parents gave informed consent for the children. A total of 874 children were enrolled and completed the anthropomorphic assessment.

### Measurement

Height and weight were measured following standard protocol (e.g. bare feet and light clothes), and the scales were recalibrated after each measurement. BMI was calculated as weight (in kg) over height (in meters and squared). The percent body fat was measured with the Futrex 6100 A/ZL body composition analyzer (Futrex, Inc, Maryland, USA) [17]. Futrex uses near infrared light to measure body fat from a single point in the human body, the middle of the bicep in the dominant arm. During operation, the analyzer sends a light beam into the bicep. The fat mass absorbs the light and the lean mass reflects it. The light absorption is measured by the analyzer to determine the percent body fat. Futrex does not require a pretest calibration before operation.

### Analysis

All analyses were conducted for boys and girls separately. First, the prevalence of overweight and obesity were determined using the standard four group classification for BMI (i.e. thin, normal, overweight, and obese) [15] and percent body fat (i.e. under-fat, normal, over-fat, and obese) [16]. The cut-off values were age- and sex-specific for BMI and percent body fat. Further, the prevalence of overweight or obesity was calculated for each age category (6–10). For girls, 6 and 7 year olds were put together because there were few girls who were 6 years old. For the comparative analyses between BMI and percent body fat, the bottom two categories were collapsed into one (e.g. thin and normal) and the top two categories into the other (e.g. overweight and obese), for both BMI and percent body fat method. The sensitivity, specificity, positive and negative predictive value, and agreement rate were calculated between the two methods using the binary variables; receiver operating characteristics (ROC) curve were generated to estimate the area under the curve [18]. Data were analyzed with SPSS (version 16), and all-tests employed were two-sided with alpha value of 0.05.

## Results

Of the sample, 70.7% (n = 618) were boys and 29.3% (n = 256) were girls; for boys, the mean age was 8.2 ± 1.3 (standard deviation) and for girls it was 8.7 ± 1.1. The girls, on average, were significantly taller (131 vs. 124 cm) and had higher weight (31.4 vs 24.9 kg) compared to the boys (p-values: <0.0001). The mean BMI for girls was also significantly higher (17.9 vs. 16.0 kg/m^2^) compared to the boys, but their mean percent body fat was significantly lower (16.3 vs. 17.3%) (p-values: <0.0001). When analyzed as continuous variables, BMI and percent body fat were positively correlated with each other (Pearson’s correlation coefficient: boys =0.71, girls =0.81) [data not shown].

**Figure 1:**
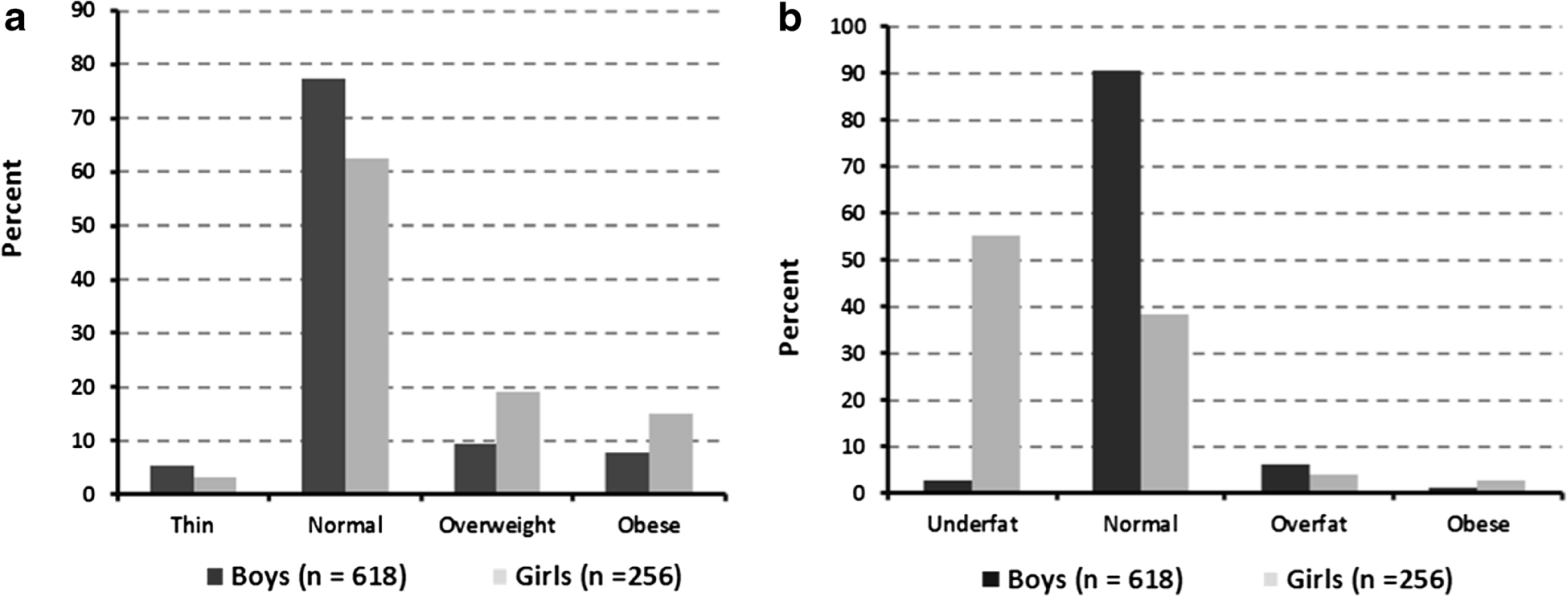
**Overweight and obesity prevalence among Saudi school children: by body mass index (BMI, 1a) and percent body fat (1b), stratified by sex.**

According to BMI, the estimates for overweight and obese prevalence were 12.4% and 9.9%, respectively. Among the boys, the prevalence estimates were 9.5% and 7.8%; among the girls the corresponding estimates were 19.1% and 15.2%. Overall, the pooled prevalence for overweight/obese was significantly higher among girls than boys (34.3% vs. 17.3%, p-value <0.0001) (Figure 1a). According to percent body fat, the over-fat and obese prevalence among boys were 6.0% and 1%, respectively; the corresponding prevalence estimates among girls were 3.9% and 2.7%. Overall, the pooled prevalence for over-fat/obese was similar between the sexes (7% vs. 6.6%, p-value 0.86) (Figure 1b).

**Figure 2:**
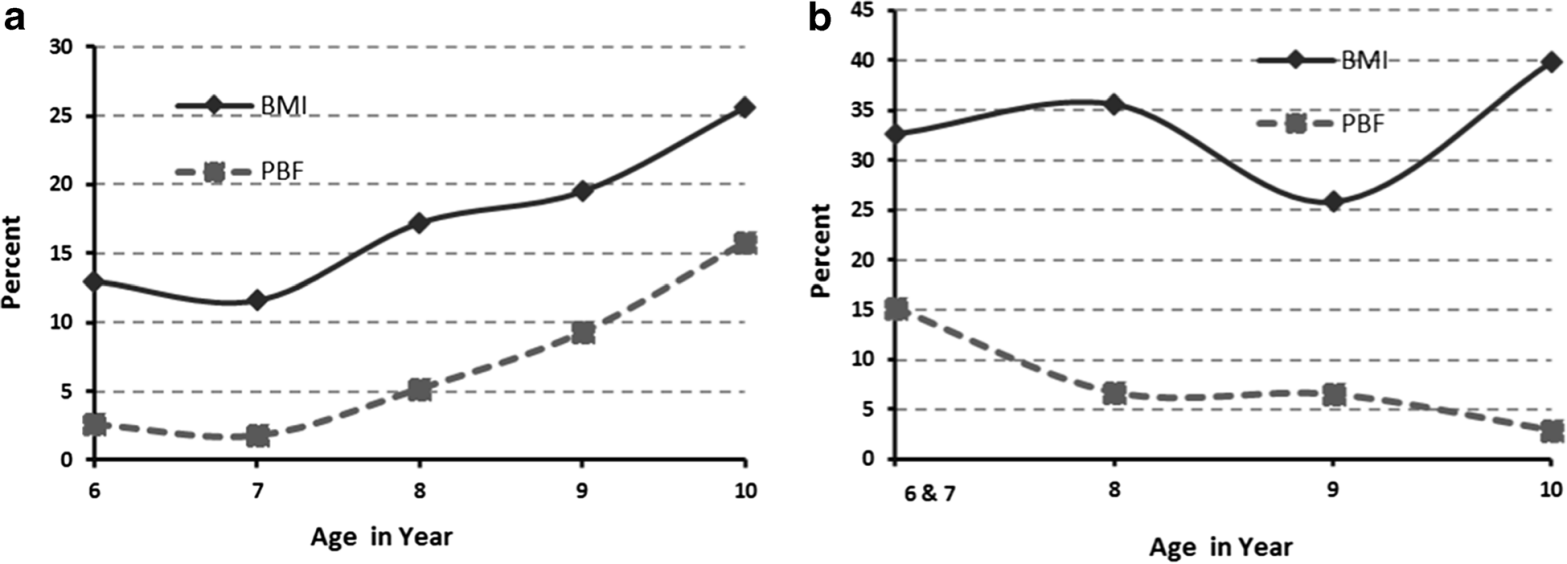
**Overweight or obesity prevalence among Saudi school children: by body mass index and percent body fat, stratified by sex and age (boys = 2a; girls = 2b).**

In both sexes, the prevalence of overweight/obese was higher using BMI than percent body fat consistently across the age groups (Figure 2a,b). Irrespective of method, for boys overweight/obese prevalence increased incrementally with age; however, in girls, there was no such pattern (Figure 2).

The sensitivity of BMI to classify overweight or obese was high in both sexes; the false positive rate compared to body fat was also high (boys = 63%, girls = 81%). The specificity of BMI was higher in boys (88%) than in girls (70%). The agreement between the BMI and percent body fat for the detection of overweight or obesity was low (boys =0.48, girls = 0.24) (Table 1).

**Table 1:** **Concordance of obesity classification using body mass index and percent body fat, stratified by sex**

**Parameter**	**Boys (n = 618)**	**Girls (n = 256)**
Sensitivity	93% (40/43)	100% (17/17)
Specificity	88% (508/575)	70% (168/239)
Positive predictive value	37% (40/107)	19% (17/88)
Negative predictive value	99% (508/511)	100% (168/168)
Agreement rate	0.48	0.24
ROC (sensitivity/1-specificity)	0.90	0.85

## Discussion

The prevalence of overweight and obese children according to BMI in this study are comparable to the results of similar studies in Saudi Arabia. For example, the overweight prevalence was 12.4% in our study, which is in the range of estimates of the previous studies (12% -24%). Similary, the obesity prevalence was 9.9%, while the range of previously reported estimates was 6% to 10% [1-3,12-14]. Our overweight estimate is seemingly low, but it is likely to reflect a higher prevalence because our study had a younger sample (6 – 10 years) while the other studies had samples with broader age ranges (6 – 18 years) – given the pattern that obesity tends to increase with age.

In our study, the prevalence of overweight and obese children are higher using BMI as opposed to percent body fat; and it is true in both sexes and in all age groups. The sensitivity of BMI for overweight or obesity diagnosis is high but the specificity is low, irrespective of sex. These findings are at odds with other studies in children that reported a low sensitivity and high specificity for BMI [19-21]. There are plausible explantions for this difference. First, we used NIR technology to assess percent body fat whereas the other studies used skinfold method. Secondly, our methods for classification of obesity were different; we used established cut-off values (WHO) as opposed to relative comparisons (overweight: >85% percentile; obese: >95% percentile). Finally, our findings could be explained by the cut-off values that we have used for classification. These cut-off values associated with each method were established in different cohorts of children, hence the difference in the methods may actually represent the differences in the underlying cohorts.

Our study has some notable strengths; it had a sufficient sample size and the sample was randomly selected. Hence, the results of the study should be generalizable to the school children in the Qassim province. Finally, our study compared two field methods for assessing obesity, and it is the first of its kind in Saudi Arabia.

We should mention some limitations of our study; our recruitment of girls was lower than boys and therefore we have an imbalanced ratio of male to female participants. Also, we sampled only one province so our results may not be generalizable to the entire country or region. NIR measures body fat at a single point on the body; hence, the estimate may vary according to how the body fat is distributed on the person. Finally, the NIR method that we used to determine percent body fat uses infrared technology but the equation it is based upon has not yet been published and validated among children.

Our study findings indicate that more children are classified as overweight or obese using BMI than the percent body fat, obtained by NIR. Since body fat composition is considered to be a more specific assessment, it is assumed that BMI is over-classifying those who may have more muscular body types. For example, most girls in our study were likely to have not reached menarche at the time of assessment (age 6–10 years), which could explain the higher estimate for BMI and lower estime for percent body fat among girls compared to boys. One way to address this issue is by correcting the cut-off values. In our study, we used the values published by WHO, but region-specific BMI cut-off values need to be determined and validated. BMI cut-off values have been established with healthy children (i.e. children were breastfed and from homes where there was no smoking or food insecurity); therefore, the cut-off values represent ideal growth standards rather than references. While food insecurity is not a problem in Saudi Arabia but the BMI cut-off values might not be applicable because of the low breastfeeding rate and high secondhand smoke exposure to the pregnant mothers in the country [22,23].

## Conclusions

The NIR technology does not have many studies to provide scientific evidence. Future studies need to be conducted to compare NIR with DEXA in order to validate its use in the field among children and adults alike.
